# Protein kinase C delta mediates Pasireotide effects in an ACTH-secreting pituitary tumor cell line

**DOI:** 10.1007/s40618-023-02117-0

**Published:** 2023-05-26

**Authors:** E. Gentilin, P. Borges De Souza, M. R. Ambrosio, M. Bondanelli, I. Gagliardi, M. C. Zatelli

**Affiliations:** 1 Bioacoustics Research Laboratory, Department of Neurosciences, University of Padua, Padua, Italy; 2 Section of Endocrinology, Geriatrics & Internal Medicine, Dept. of Medical Sciences, University of Ferrara, Ferrara, Italy

**Keywords:** Pasireotide, ACTH, POMC, PRKCD, Corticotroph tumors

## Abstract

**Purpose:**

Clinical control of corticotroph tumors is difficult to achieve since they usually persist or relapse after surgery. Pasireotide is approved to treat patients with Cushing’s disease for whom surgical therapy is not an option. However, Pasireotide seems to be effective only in a sub-set of patients, highlighting the importance to find a response marker to this approach. Recent studies demonstrated that the delta isoform of protein kinase C (PRKCD) controls viability and cell cycle progression of an in vitro model of ACTH-secreting pituitary tumor, the AtT-20/D16v-F2 cells. This study aims at exploring the possible PRKCD role in mediating Pasireotide effects.

**Methods:**

It was assessed cell viability, POMC expression and ACTH secretion in AtT20/D16v-F2 cells over- or under-expressing PRKCD.

**Results:**

We found that Pasireotide significantly reduces AtT20/D16v-F2 cell viability, POMC expression and ACTH secretion. In addition, Pasireotide reduces miR-26a expression. PRKCD silencing decreases AtT20/D16v-F2 cell sensitivity to Pasireotide treatment; on the contrary, PRKCD overexpression increases the inhibitory effects of Pasireotide on cell viability and ACTH secretion.

**Conclusion:**

Our results provide new insights into potential PRKCD contribution in Pasireotide mechanism of action and suggest that PRKCD might be a possible marker of therapeutic response in ACTH-secreting pituitary tumors.

## Introduction

Adrenocorticotropic Hormone (ACTH)-secreting pituitary tumors are pituitary neoplasms characterized by an excessive ACTH release. The latter is associated with chronic endogenous hypercortisolism, a medical condition called Cushing’s disease (CD), a grim endocrine disease related with increased morbidity and mortality [1]. CD therapy is one of the most challenging of all endocrine diseases. Surgery represents the main therapeutic approach, but one third of patients face disease persistence or recurrence [2]. To date, medical therapy plays a very important role in CD clinical control. Indeed, it is employed in various conditions, such as presurgical treatment aimed at reducing cortisol levels before surgery, postsurgical second-line treatment, first line treatment in the absence/refusal of surgical indication and radiotherapy combined treatment [1]. Pasireotide is the only approved pituitary-directed drug for adult patient with CD for whom surgical therapy is not a therapeutic option. Pasireotide is a somatostatin (SST) receptor (SSTR) ligand that binds with a high affinity four out of five SSTRs (SSTR5 > SSTR2 > SSTR3 > SSTR1) [3]. Despite its efficacy has been proven on clinical grounds [4], Pasireotide is able to normalize cortisol secretion only in ~ 40% of CD patients [5]. Thus, exploiting Pasireotide mechanism of action may be important to find new pathways to enhance the inhibitory effects of this drug on ACTH secretion. In addition, the identification of predictive markers of response to Pasireotide is crucial to improve patient management. Recent studies have demonstrated that several proteins belonging to protein kinase C family (PRKC) are involved in the control of ACTH secretion [6]. Among PRKC proteins, the delta isoform (PRKCD) controls viability and cell cycle progression of an "in vitro" model of ACTH-secreting pituitary tumor, the AtT-20/D16v-F2 cells [6].

This study aims at exploring PRKCD role in mediating Pasireotide effects by assessing cell viability, POMC expression and ACTH secretion in AtT20/D16v-F2 cells over- or under- expressing PRKCD, in order to identify a possible marker of Pasireotide sensitivity.

## Materials and methods

### Cell cultures

The mouse ACTH-secreting pituitary tumor cell line, AtT-20/D16v-F2 (ATCC CRL-1795, American Type Culture Collection, Manassas, Virginia, USA), was maintained at 37 °C, 5% CO_2_ in DMEM high glucose (Invitrogen, Carlsbad, California, USA) supplemented with 10% horse serum (LGC Standards, Milan, Italy) and antibiotic/antimycotic (EuroClone, Milan, Italy). The cell line previously obtained stably transfecting AtT-20/D16v-F2 cells with PRKCD shRNA and the cell line AtT-20/D16v-F2 stably transfected with scrambled oligonucleotides were maintained at the same conditions of parental cells [6].

### Transfection

AtT-20/D16v-F2 cells were seeded at the concentration of 150.000 cells/well in 6-well plates containing 2 ml of medium. After 24 h, the cells were transfected with PRKCD-pCMV6-ENTRY vector (OriGene, Rockville, MD, USA) using the lipofectamine LTX DNA transfection reagents (Thermo Fisher Scientific, Waltham, MA, USA), according to the manufacturer's protocol. Briefly, the cells were transfected using two combined solutions. The first one was made using 5 μg of DNA, 135 μl of Opti–MEM^®^ Medium and 5 μl of PLUS Reagent and the second one using 135 μl of Opti-MEM^®^ Medium and 15 μl of lipofectamine LTX. The pCMV6-ENTRY vector (OriGene) was used as control. Stably transfected clones were selected by incubation in medium containing geneticin 0.5 mg/ml (Thermo Fisher Scientific). All the experiments involving transfected cells were performed using two different stable clones in order to increase the reliability of the results.

### Cell viability assay

9.000 cells/well were seeded in 96-well white plates and treated with 10 nM Pasireotide for 48 h. Exposure time and drug concentration were in line with previous findings showing that Pasireotide inhibits ACTH secretion with the maximum response achieved after 48–96 h with 1–10 nM concentration [7]. At the end of incubation time, cell viability was assessed by the ATPlite assay (PerkinElmer, Waltham, Massachusetts, USA), as previously reported [8]. Results are expressed as mean value ± Standard Error of the Mean (SEM) percent viable cell number vs. control cells from three independent experiments in 6 replicates.

### Western blot

The cells were seeded at a cell density of 500.000 cells in 60-mm plates. After 24 h, cells were treated with or without 10 nM Pasireotide for 48 h. At the end of the incubation time, proteins were isolated and quantified by the BCA Protein Assay Reagent Kit (Pierce Biotechnology, Inc, Rockford, Illinois, USA). 30 μg of proteins were separated by SDS-PAGE and then transferred by electroblot to nitrocellulose membranes (Schleicher & Schuell Italia SRL, Milan, Italy). Blots were first exposed overnight at 4 °C to 1/200 rabbit anti-proopiomelanocortin (POMC) antibody, and then to 1/2000 rabbit horseradish peroxidase-conjugated secondary antibodies (Dako Italia, Milan, Italy) for 1 h at room temperature. The membranes were visualized using the enhanced chemiluminescence Western blotting detection reagents (GE Healthcare Europe GmbH, Milan, Italy). At least three independent experiments were performed and ImageJ was used to relatively quantify protein bands from Western blots (https://imagej.nih.gov).

### ACTH secretion

ACTH secretion was evaluated by ELISA as previously reported [9]. Results were normalized by viable cell number, as determined by the ATPlite assay. Results are expressed as the mean value ± SEM percent ACTH concentration vs. control cells.

### Real-time PCR analysis

cDNA was synthesized using the TaqMan MicroRNA reverse transcription kit (Applied Biosystems, Monza, Italy) and miRNA gene-specific primer sets supplied with the TaqMan MicroRNA Assays hsa-miR-26a PN4373070 (Applied Biosystems). The levels of miR26a were evaluated by relative quantitative real-time PCR using the Applied Biosystems 7700 ABI Prism thermal cycler (Applied Biosystems) as previously reported [10]. Results are expressed as mean value ± SEM percent miRNA expression vs. control cells from two independent experiments in three replicates.

### Statistical analysis

For multiple comparison analysis, statistical significance of parametric data was tested by ANOVA, while statistical significance of non-parametric data was tested by Kruskal–Wallis test with Mann–Whitney as post hoc test. For comparison of two groups, the *t* test for parametric data and the Mann Whitney test for non-parametric data were employed.

## Results

### Effects of Pasireotide on AtT-20/D16v-F2 cells

**Fig. 1:**
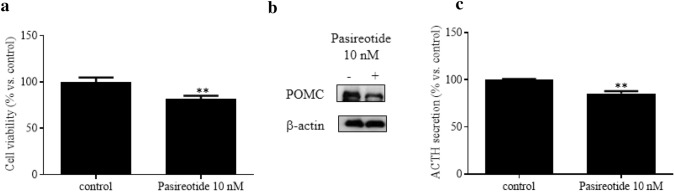
Pasireotide action on viability, POMC expression and ACTH secretion of AtT20/D16v-F2 cells. Cells were incubated for 48 h in culture medium supplemented with or without Pasireotide 10 nM in at least 3 replicates. **a** Cell viability is expressed as mean value percent ± standard error of the mean (SEM) *vs.* control cells. ** = *p* < 0.01 *vs.* control cells. **b** Representative Western blot showing POMC expression. Beta-actin was used as loading control. **c** ACTH secretion is expressed as mean value percent ± SEM *vs.* control cells. ** = *p* < 0.01 *vs.* control

First, the effects of Pasireotide were evaluated in the AtT-20/D16v-F2 cells. Pasireotide at the concentration of 10 nM significantly reduced cell viability by ~ 20% (*p* < 0.01 vs. control; Fig. 1A), POMC expression by ~ 30% (Fig. 1B) and ACTH secretion by 16% as compared to control cells (p < 0.01 vs. control; Fig. 1C). In addition, the effects of Pasireotide on miR-26a expression were analyzed.

**Fig. 2:**
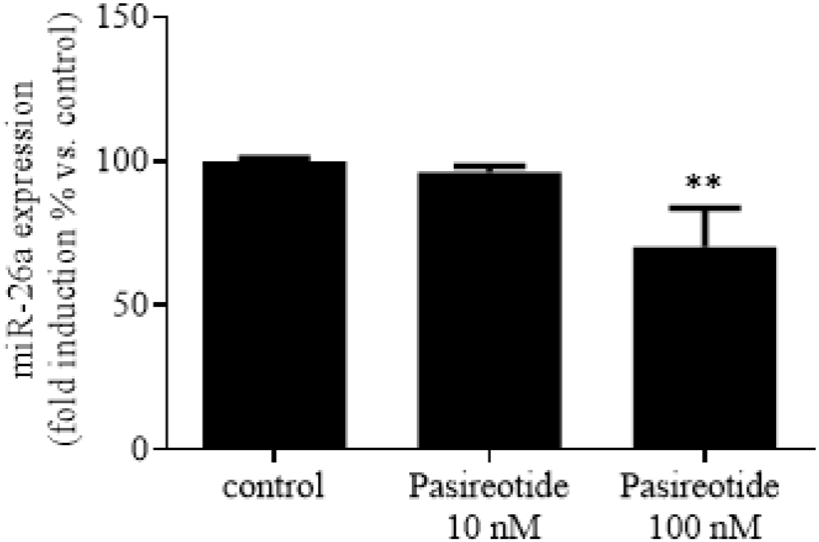
Pasireotide effects on miR-26 expression in AtT20/D16v-F2 cells. Cells were incubated for 48 h in culture medium supplemented with or without with increasing concentrations of Pasireotide (10 and 100 nM). miR-26a expression is expressed as mean value percent ± SEM *vs.* control cells in at least 3 replicates. ** = *p* < 0.01 *vs.* control

As reported in Fig. 2, increasing concentrations of Pasireotide on one hand tended to increase PRKCD protein expression (Fig. 2A), and on the other hand significantly reduced miR-26a expression (~ 30%; *p* < 0.01 vs. control; Fig. 2B).

### Effects of PRKCD modulation on Pasireotide activity

**Fig. 3:**
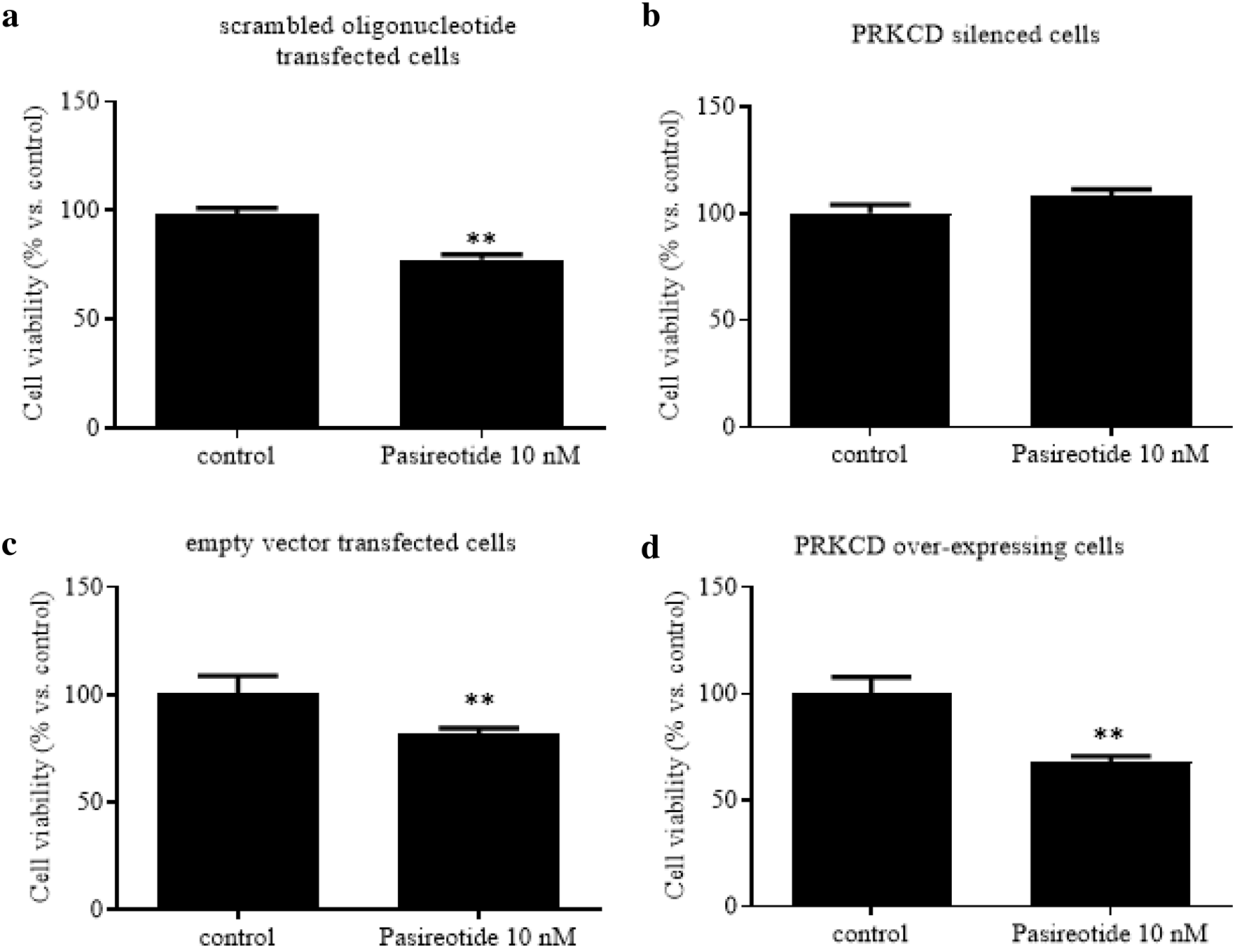
PRKCD silencing and overexpression in AtT20/D16v-F2 cells: effects on cell viability under Pasireotide treatment. Cells were incubated for 48 h in culture medium supplemented with or without Pasireotide 10 nM in at least three replicates. Scrambled oligonucleotide transfected cells: control for PRKCD-silenced cells. Empty vector transfected cells: control for PRKCD over-expressing cells. Cell viability is expressed as mean value percent ± SEM *vs.* control cells. ** = *p* < 0.01 *vs* control

We then investigated whether the effects of Pasireotide are mediated by PRKCD. To this aim, first PRKCD was stably silenced or over-expressed in AtT-20/D16v-F2 cells, and then the stably transfected cells were treated with or without Pasireotide 10 nM for 48 h. We also generated stable control cell lines, which were treated in the same way. At the end of the incubation time, the cells were assessed for cell viability, POMC expression and ACTH secretion. Regarding cell viability, as shown in Fig. 3, Pasireotide significantly reduced viability of scrambled oligonucleotide transfected cells by ~ 20% as compared to controls (*p* < 0.01 vs. control; Fig. 3A), while it did not affect viability of PRKCD-silenced cells (Fig. 3B). In addition, Pasireotide significantly reduced viability of empty vector transfected cells by ~ 20% (*p* < 0.01 vs. control; Fig. 3C) and of PRKCD over-expressing cells by 32% (*p* < 0.01 vs. control; Fig. 3D).

**Fig. 4:**
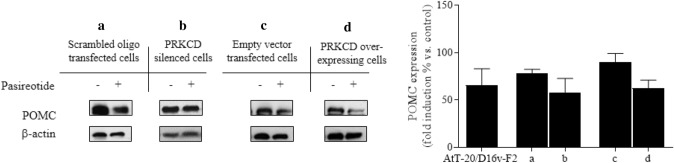
Modulation of PRKCD levels in AtT20/D16v-F2 cells and Pasireotide effects on POMC expression. Cells were incubated for 48 h in culture medium supplemented with or without Pasireotide 10 nM in at least 3 replicates. POMC expression quantification. **a** Scrambled oligonucleotide transfected cells, **b** PRKCD-silenced cells, **c** empty vector transfected cells, **d** PRKCD over-expressing cells

As concerns POMC expression (Fig. 4), Pasireotide reduced this parameter in all cell clones nearly to the same extent, without reaching statistical significance.

**Fig. 5:**
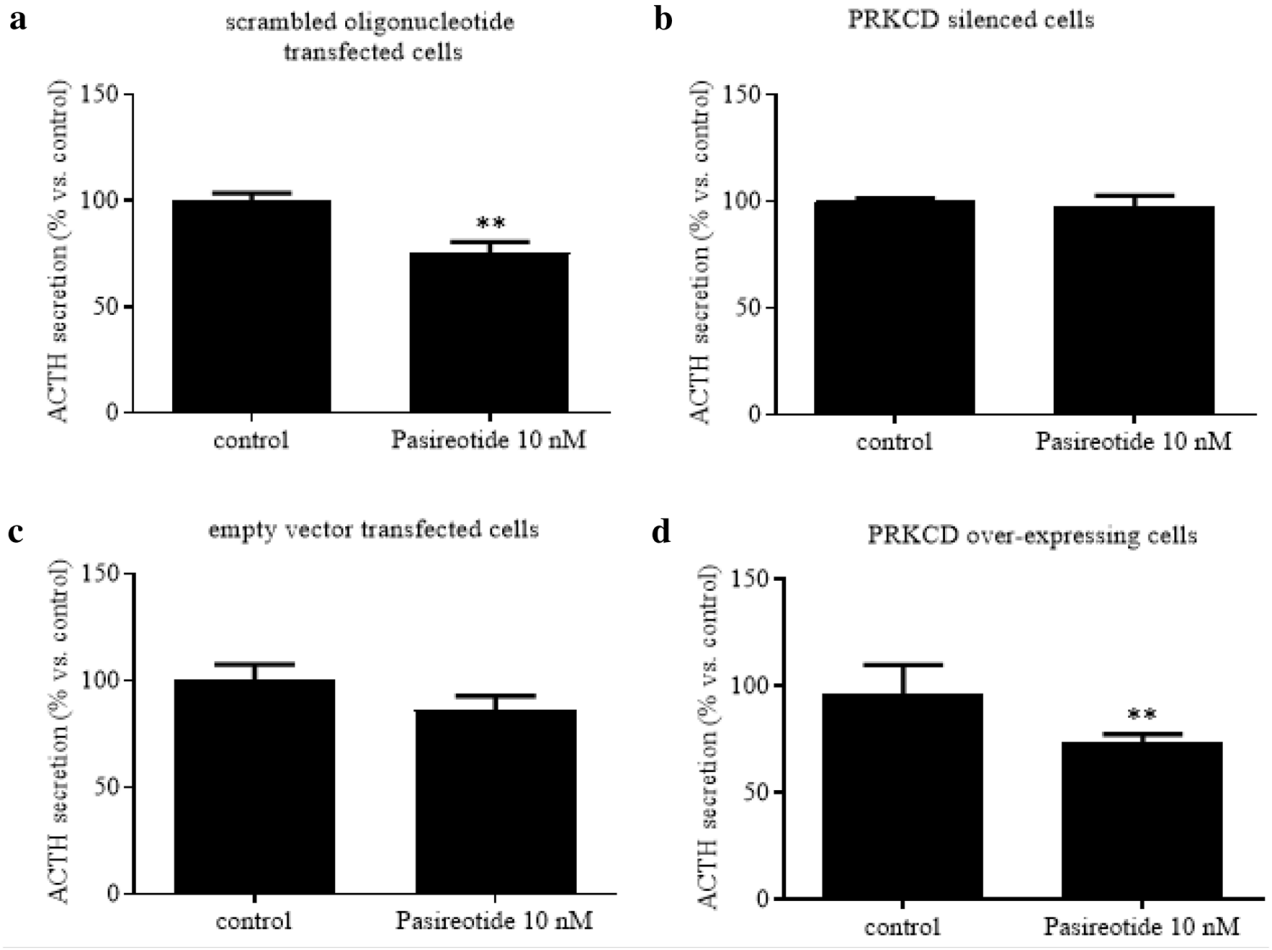
PRKCD silencing and overexpression in AtT20/D16v-F2 cells: effects on ACTH secretion under Pasireotide treatment. Cells were incubated for 48 h in culture medium supplemented with or without Pasireotide 10 nM in at least three replicates. Scrambled oligonucleotide transfected cells: control for PRKCD-silenced cells. Empty vector transfected cells: control for PRKCD over-expressing cells. ACTH secretion was expressed as mean value percent ± SEM *vs.* control cells. ** = *p* < 0.01 *vs.* control

With respect to ACTH secretion (Fig. 5), Pasireotide significantly reduced ACTH secretion by scrambled oligonucleotide transfected cells by 24% (*p* < 0.01 vs. control; Fig. 5A), while it did not affect ACTH secretion by PRKCD-silenced cells as compared to controls (Fig. 5B). In addition, Pasireotide reduced ACTH secretion of empty vector transfected cells by 14% without reaching statistical significance (Fig. 5C) but significantly reduced ACTH secretion by PRKCD over-expressing cells by 27% (*p* < 0.01 vs. control; Fig. 5D).

## Discussion

Pasireotide is a multiligand SSTR agonist whose efficacy in reducing cortisol both “in vivo” and “in vitro” led to its approval as pharmacological treatment of CD [11]. Indeed, it has been reported that treatment of CD patients with subcutaneous Pasireotide for 12 months decreases ACTH plasma levels, normalizes urinary free cortisol in up to 25% of patients and reduces tumor size [11–16]. In addition to the approved twice-daily subcutaneous formulation, an intramuscular long-acting release (LAR) formulation adjusted for once-monthly administration has been developed. The results from the first phase III trial evaluating intramuscular Pasireotide LAR in CD patients showed a normalization of mean urinary free cortisol in about 40% of patients [17]. In addition, Pasireotide LAR was also demonstrated to be able to reduce plasma ACTH in patients with Nelson syndrome [18, 19]. In dogs, Pasireotide was effective in controlling CD reducing ACTH secretion and POMC transcription [20]. And again, in rats, Pasireotide inhibited ACTH secretion [21]. In vitro studies showed that the drug reduces ACTH secretion in cultures of human ACTH-secreting pituitary tumors [22] and impairs ACTH secretion in the murine ACTH-secreting pituitary tumor cell line AtT-20 [23]. In addition, beyond the effects on ACTH secretion, Pasireotide also affected cell growth of pituitary corticotroph tumors both in vitro and in vivo [8, 24].

In this study, using the AtT-20/D16v-F2 cells, the F2 subclone of original AtT-20 cells, we confirmed that, in keeping with previously published evidence, Pasireotide significantly reduces corticotroph cell viability and ACTH release.

CD still persists uncontrolled in some Pasireotide-treated patients, and even among those showing a clinical and biochemical remission resistance can develop. Therefore, the identification of the mechanism of action of Pasireotide in order to identify a possible marker of Pasireotide sensitivity is crucial to improve patient management. The latter represents an important issue, especially bearing in mind that Pasireotide treatment is associated with the development of hyperglycemia of different degrees in most patients, which needs attention and a suitable diabetes control plan [14, 16, 17]. Since PRKCD is down-regulated in human ACTH-secreting pituitary tumors and it was proven to be involved in the control of cell viability as well as POMC and ACTH expression in AtT20/D16v-F2 cells [6, 10], we explored PRKCD role in mediating Pasireotide effects in AtT20/D16v-F2 cells over- or under-expressing PRKCD. PRKCD is a serine/threonine kinase that regulates many physiopathological processes through the phosphorylation of target proteins [25, 26]. The relevance of PRKCD in growth regulation of ACTH-secreting pituitary tumor cells was firstly highlighted by a study demonstrating that high PRKCD levels, under the control of miR-26a, delay cell cycle in G1 phase [10]. These results were later strengthen by the evidence that PRKCD per se influences the mechanisms regulating pituitary adenoma cell viability [6]. In addition, it was shown that PRKCD silencing increases POMC and ACTH expression demonstrating that not only PRKCD exhibits tumor suppressing activities, but also regulates ACTH secretory mechanisms [6].

We here show that Pasireotide reduces the levels of miR-26a, one of the negative regulators of PRKCD expression. Considering that PRKCD plays an important role in several metabolic and signaling pathways in corticotroph tumors, it is plausible that PRKCD could represent an important target for drug activity. The evidence that PRKCD is also activated by the SST analogue TT-232 [27], supports this hypothesis suggesting that PRKCD may generally represent a crucial signaling protein of SSTR ligands mechanism of action.

On the basis of these promising results, we investigated whether the modulation of PRKCD expression influences Pasireotide effects on AtT20/D16v-F2 cells. We observed that PRKCD down-regulation completely abolishes Pasireotide effects on cell viability and ACTH secretion, while PRKCD overexpression enhances Pasireotide effects on these parameters. These results could explain conflicting reports in the literature regarding the efficacy of Pasireotide in terms of ACTH secretion and tumor restrain. Many reports support the use of Pasireotide due to its efficacy in controlling these parameters [22, 23, 28–30]. However, Pasireotide did not seem just as effective in other studies [31]. The lack of efficacy of Pasireotide shown in some corticotropinomas strongly suggests that additional mechanisms besides the already known SSTR profile are necessary to achieve full drug effectiveness.

In our study, POMC expression at protein level did not seem to be influenced by Pasireotide or by PRKCD levels. Therefore, our data suggest that PRKCD may mediate Pasireotide effects on ACTH secretion rather than on POMC expression. Previous reports showed that Pasireotide inhibits POMC promoter activity and POMC mRNA expression in AtT20 cells mainly via SSTR2 [20, 31]. On the contrary, our data show the lack of a significant effect on POMC protein levels by Pasireotide, suggesting that the reduction in ACTH levels is mainly due to an antisecretory effect of Pasireotide in our model, in keeping with the evidence that AtT20 cells mainly express SSTR5 [32].

Finally, these results were obtained using different transfected stable cell lines, and it is fundamental to underline that the results obtained in control cells were comparable to those obtained in the parental cell line. The only exception was observed analyzing ACTH secretion in empty vector transfected cells. In these cells, treatment with Pasireotide reduced ACTH secretion without reaching statistical significance as observed in parental cells. DNA transfection is considered the tool of choice for investigating the role of genes/proteins, but it may be associated with pitfalls, off-target effects and artifacts [33]. Therefore, slight variations are expected as far as there is no change of scientific meaning. In addition, all the comparisons were performed focusing on the differences between each control cell line with its matching PRKCD transfected cell clone.

Our results, therefore, indicate that PRKCD levels affect Pasireotide efficacy in controlling corticotroph growth and function. Recent studies on corticotropinomas focusing on ubiquitin specific protease 8 (USP8) show that the USP8 mutational state appears to be related to SSTR5 expression [34], but a correlation between USP8 mutation or SSTR5 expression and Pasireotide response remains to be confirmed, even if promising results have been recently presented [35]. Therefore, there is still a strong need for a marker of Pasireotide efficacy in controlling CD. Our data suggest that PRKCD levels may predict Pasireotide efficacy. However, this hypothesis needs to be confirmed in further studies employing human pituitary tumor tissues.

In conclusion, our data demonstrate that PRKCD mediates the antisecretory effects of Pasireotide on a murine corticotroph cells line and candidate PRKCD as a putative marker of Pasireotide efficacy. Thus, it is clear that medical trend is moving from “trial and error” process to precision medicine based on molecular analysis to avoid delayed cures and possible side effects.

## Data Availability

Data are available upon request to the corresponding author.
